# Synthesis and Characterization of Acetic Acid-Doped Polyaniline and Polyaniline–Chitosan Composite

**DOI:** 10.3390/biomimetics4010015

**Published:** 2019-02-11

**Authors:** Bianca Rae Pasela, Acelle Pearl Castillo, Rhenish Simon, Maria Teresa Pulido, Haidee Mana-ay, Ma. Roxan Abiquibil, Rhys Montecillo, Kanjana Thumanu, Doebner von Tumacder, Kathrina Lois Taaca

**Affiliations:** 1 Department of Physics, Mapúa University Intramuros, Manila 1002, Philippines; brpasela@gmail.com (B.R.P.); castilloacelle@gmail.com (A.P.C.); mariateresapulido@gmail.com (M.T.P.); 2 Department of Physical Sciences and Mathematics, University of the Philippines Manila, Manila 1000, Philippines; rcsimon@up.edu.ph; 3 Department of Physics, Silliman University, Dumaguete 6200, Philippines; manaay88@gmail.com (H.M.); roxanabiquibil@gmail.com (M.R.A.); 4 Department of Physics and Geology, Negros Oriental State University, Dumaguete 6200, Philippines; montecillo.rhys@gmail.com; 5 Synchrotron Light Research Institute (Public Organization), Nakhon Ratchasima 30000, Thailand; kanjanat@slri.or.th; 6 Department of Mining, Metallurgical and Materials Engineering, College of Engineering, University of the Philippines Diliman, Quezon 1101, Philippines

**Keywords:** polyaniline, chitosan, composite, emeraldine, trypan blue assay

## Abstract

Polyaniline–chitosan (PAni–Cs) composite films were synthesized using a solution casting method with varying PAni concentrations. Polyaniline powders used in the composite synthesis were polymerized using acetic acid as the dopant media. Raman spectroscopy revealed that the PAni powders synthesized using hydrochloric acid and acetic acid did not exhibit significant difference to the chemical features of PAni, implying that PAni was formed in varying concentrations of the dopant media. The presence of agglomerated particles on the surface of the Cs composite, which may have been due to the presence of PAni powders, was observed with scanning electron microscope–energy dispersive X-ray spectroscopy (SEM–EDX). Ultraviolet–visible (UV–Vis) spectroscopy further showed the interaction of PAni with Cs where the Cs characteristic peak shifted to a higher wavelength. Cell viability assay also revealed that the synthesized PAni–Cs composites were nontoxic and may be utilized for future biomedical applications.

## 1. Introduction

A biomaterial is primarily designed to serve as a mechanical and physiological substitute to a human body part, without the threat of having a hostile reaction in the system [1]. This nondrug substance is considered an essential element in improving the human health and quality of life since it is found to be suitable for inclusions to human systems [2,3]. Biomaterials are generally classified into metals, ceramics, polymers, and composites. All biomaterials are used in restoring function and facilitating healing application by supporting, enhancing, or replacing a damaged tissue or a biological function in a human body [4,5]. Biomaterials are generally used in medical fields such as diagnostics, medical supplies, therapeutic treatments, and regenerative medicine [3].

Polymers offer a versatility unmatched by metals and ceramics. The wide spectrum of physical, mechanical, and chemical properties provided by polymers has fueled the extensive research, development, and applications of polymeric biomaterials [3]. Polymers are composed of a large number of repeating units that can be bonded together to form a three-dimensional network and in biological systems present a diverse range of functions [6]. Polymers, however, are often combined with metal, ceramic, or other polymers to form composites that can be easily modified, with higher biocompatibility, than most synthetic polymers [1,7]. Moreover, polymer composites, have enhanced optical, thermal, mechanical, magnetic, and optoelectronic properties because of the addition of fillers, inorganic or organic, which are used to complement the properties of the polymer matrix [1,6]. These fillers often have properties, such as a large surface area, high surface reactivity, excellent thermal stability, high mechanical strength, flexibility, or good processability. The combination of properties of the filler and matrix in a polymer composite have found wide use in diversified fields like sensing, solar cell, biomedicine, and biotechnology [6]. Aside from their desirable properties, polymers are cheaper than other materials and can help in the advancement of the biomedical field [7].

Chitosan (Cs) is a natural biopolymer derived from chitin, composed of –OH and –NH_2_ functional groups in its acetyl and *N*-acetyl glucosamine units [1,8]. It has been utilized by several research studies towards the advancement of different biomedical areas, such as wound healing, drug delivery, and tissue engineering [8]. However, Cs-based films exhibit poor mechanical functionality, which may limit their applications. Thus, multiple approaches have been employed to improve the barrier and mechanical performance of these films. In recent years, a substantial amount of research has dealt with the blending of chitosan and various polymers [9]. These studies have developed several Cs-based systems with advanced properties (depending on the intended application). Chitosan-based systems include those capable of removing heavy metals and dyes (cationic, anionic, methylene blue, and methyl orange) such as graphene oxide–chitosan and magnetic chitosan, as well as other inorganic wastes such as magnetic Cs–biochar, amine-grafted Cs–polymethyl methacrylate (PMMA), and chitosan–hexadecylamine [10].

Polyaniline (PAni), on the other hand, is a conducting polymer that has been the focus for a variety of applications in the field of materials science [11]. It is considered to be one of the most attractive conducting polymers due to its high conductivity and good stability [12]. In addition, PAni finds applications in such fields as light-emitting diodes, plastic batteries, solid-state sensors, rechargeable batteries, antistatic and anticorrosion coating, microelectronics, and electromagnetic shielding [12,13]. The suitability of PAni for such applications is possible due to its semiflexible, low-cost, and environmentally stable rod-like features [13]. Polyaniline can be easily prepared using various methods and can also be modified to change its surface properties [14]. This, therefore, makes it a well-suited polymer for applications like tissue engineering and biosensors.

Conducting-polymers like PAni are promising materials in engineering tissues due to a combination of features such as intrinsic electrical conductivity and easy preparation [14]. However, PAni, in its powdered form, is cytotoxic possibly due to the presence of low-molecular weight impurities [15]. Studies have already presented methods for preparing high-purity PAni [15]. One of these is the fabrication of PAni films combined with other polymers [16]. The modification of PAni with chitosan was seen to be a useful approach in developing environmental remediation applications [11]. Moreover, with the well-known biocompatibility of chitosan, a blend film made of PAni and Cs may possess advanced biological properties essential for biomedical applications.

In recent years, synthesis of a PAni–Cs composite has been an important area of study because of the excellent suitability of both polymers for numerous applications. The composite was synthesized using an in situ chemical oxidation process for biosensing applications [17]. This study observed a strong interaction between PAni and Cs where the composite provided better degradation properties at higher temperatures and good conductivity due to its smooth morphology compared to chitosan alone [17]. Another study developed the composite using an in situ electropolymerization method [18]. Smooth and granular morphologies of the composite were observed, although it was revealed in this study that the composite with granular porous surfaces is more suitable for immobilization of biomolecules for glucose sensing applications [18]. A PAni–Cs composite can also be formed using a solution casting approach [19]. Blend films with varying PAni content were observed to determine thermal, mechanical, and electrical conductivity properties. The study revealed that the blend films have better degradation stability at higher temperatures than pure chitosan, similar to that reported in [17]. The effect of PAni was significantly observed in the mechanical property of the blend films. The tensile strength of the films was observed to be higher at blends with 20–40 wt % of PAni content. This implied that the blend films with PAni contents not within the range (10 and 50 wt %) were more brittle and more difficult to fabricate. The addition of PAni showed that there is an optimal amount to form a ductile blend film. On the other hand, an increase in PAni content increased electrical conductivity. The effect of a hydrochloric acid (HCl) dopant on the mechanical and electrical conductivity of the composite was also investigated in this study [19]. It was observed that the ductility and the electrical conductivity of the films dramatically decreased with a higher concentration of acid dopant, due to the high acid strength of HCl and the long acid doping treatment time [19].

In previous studies, PAni–Cs composites have been developed using different synthesis approaches with HCl as the main acid dopant. This study, on the other hand, aimed to develop a PAni–Cs composite using a solution casting method. The study utilized PAni powders synthesized using acetic acid (CH_3_COOH) as the acidic medium. The effect of varying amounts of PAni powders to the morphology, chemical structure, and cell viability properties of Cs were also investigated in this study to determine the feasibility of a PAni–Cs composite that could be applied for healing wounds.

## 2. Materials and Methods

### 2.1. Materials

Chitosan powder from shrimp shells (C3646-25G, DD ≥ 75%) was purchased from Sigma-Aldrich (St. Louis, MO, USA), glacial acetic acid (CH_3_COOH, ACS Reagent Grade 281000ACS) was procured from Pharmco-Aaper (Brookfield, CT, USA), and glycerine was locally sourced (Quezon City, Philippines). Aniline (An), ammonium persulfate (APS), methanol, hydrochloric acid (HCl), and sodium hydroxide (NaOH) reagents were provided by the chemistry laboratory of Silliman University. All precursors and reagents were used as received and no pretreatment was done prior to the experiment. Distilled water (H_2_O) was used all throughout the experiment.

### 2.2. Preparation of PAni Powders

Pure An was prepared with HCl and CH_3_COOH as the acid dopant media. Varying amounts of acid dopants were combined with 5.48 M An solution for the polymerization process. The concentrations of each treatment are summarized in Table 1. About 4 g of APS were also mixed with each treatment as an oxidizing agent. The mixture was kept under constant stirring in an ice bath until greenish-black precipitates began to form. The polymerized powders were then filtered, washed, and neutralized with distilled H_2_O, NaOH, and methanol. Samples were air-dried and stored in a desiccator.

### 2.3. Synthesis of PAni–Cs Composite

The PAni–Cs composites were prepared via a solution casting method. A solution of 1 wt % of chitosan (Cs) and 90% CH_3_COOH was mixed and stirred at 380 rpm for 2 h at room temperature (RT). Meanwhile, 0.5% (*w*/*v*) PAni powders were dispersed in distilled H_2_O and stirred at 380 rpm until complete dissolution. The PAni suspension was then mixed with the chitosan mixture. The desired ratios of PAni–Cs (1:10 and 1:1) were stirred for an additional 30 min. About 0.8 mL glycerine was added to each mixture as plasticizer while stirring. The prepared solutions were placed in polystyrene Petri dishes and air-dried for approximately 7–10 days. Post-treatment of the composites was done by soaking the samples in an isopropyl alcohol solution. The composite samples were cut into 1 × 1 cm films prior to characterization.

### 2.4. Characterization of PAni Powder and PAni–Cs Composites

The chemical features (vibrational, rotational, and other low-frequency modes) of the synthesized PAni powders were investigated using a homemade micro-Raman spectroscopy (DU401_BVF, National Institute of Physics, University of the Philippines Diliman, Quezon City, Philippines) [20]. The Raman system is composed of 5X, 10X and 100X Mitutoyo plan apochromatic long working distance objectives, an iHR 550 spectrometer from Horiba Jobin Yvon, Synapse 1024x128 FIVS CCD detector from Horiba Jobin Yvon Inc., Symphony Ge detector from Horiba Jobin Yvon Inc., and Ventus 532nm laser from Laser Quantum. Powders were analyzed under a laser light source with a wavelength of 780 nm and exposure time of 30 s. The surface morphology of PAni–Cs composites was evaluated by scanning electron microscopy–energy dispersive X-ray spectroscopy (SEM–EDX) using a SU1510 electron microscope (Sigmatech, Inc., Muntinlupa City, Philippines). The composite films were observed using an accelerating voltage of 5.00 kV, with a working distance of 18.10 mm and a magnification range of 500–1500×. The interaction of PAni and Cs was observed in absorbance mode using a UV–Vis spectrophotometer (UVmini-1240, Shimadzu, Cavite, Philippines) at a wavelength range of 190–1100 nm. The wavelength of the light source was 340 nm. Polyaniline–chitosan samples were also characterized using attenuated total reflectance (ATR) mode. Spectral data were collected in the BL 4.1 infrared spectroscopy and imaging beamline at the Synchrotron Light Research Institute (SLRI). Spectra of each sample were acquired with a synchrotron radiation-based Fourier-transform infrared (SR-FTIR) composed of a Bruker Tensor 27 spectrometer (Globar source) and coupled with an infrared microscope (Hyperion 2000, Bruker Optik GmbH, Ettlin-Gen, Germany) using a 20× ATR objective lens with a mercury–cadmium–telluride (MCT) detector cooled using liquid nitrogen. The measurements were performed in ATR mode, ranging from 4000 to 800 cm^−1^, with an aperture size of 20 × 20 µm^2^ and a spectral resolution of 4 cm^−1^ with 64 scans co-added. Spectral acquisition and instrument control were performed using OPUS 7.5 (Bruker Optik GmbH) software [21].

### 2.5. Cell Viability Assay

The cell viability assay of lymphocyte cells on PAni–Cs films was conducted in the Biological Research Services and Laboratory of the Natural Science Research Institute in the University of the Philippines Diliman.

#### 2.5.1. Preparation of the Lymphocyte Culture

Lymphocyte viability was assessed by trypan blue assay and lymphocytes were collected in a green-top vacutainer tube. This was mixed with an equal volume of Dulbecco’s phosphate-buffered saline (PBS). Approximately 4 mL of the diluted blood was carefully overlaid onto 3 mL of Ficoll-Paque in each of the three centrifuge tubes, and centrifuged at 2000 rpm for 30 min at RT. The cloudy buffy coat layers of each tube were transferred to a fresh centrifuge tube with the clear plasma layer being discarded from each tube. The cells of the transferred cloudy layer were added to three volumes of Dulbecco’s PBS. This tube was then inverted several times to wash the lymphocytes thoroughly. The supernatant of the mixture was separated via centrifuging at 1000 rpm for 15 min at RT. An additional 8 mL of Dulbecco’s PBS was added to the resuspended pellet after the supernatant was discarded. After the tube was inverted several times to wash the cells, final centrifugation was performed at 1000 rpm for 10 min. The final supernatant was removed, and supplemented with RPMI 1640 media containing fetal bovine serum, penicillin–streptomycin, and amphotericin B, so that the final cell density reached 2 × 10^6^ cells/mL. The final pellet was resuspended, and the culture was incubated at 37 °C for 1 h [1,22].

#### 2.5.2. Cell Viability Assay of Lymphocytes on PAni–Cs Composites

##### Preparation of Test Samples

The composite film samples were cut from 1.0 cm × 1.0 cm into 0.5 cm × 0.5 cm squares and were UV-irradiated for at least 30 min prior to the assay. Each of the films was immersed in a microcentrifuge tube filled with 0.5 mL of RPMI supplement. These tubes were incubated at 37 °C for 24 h. Each extract was then transferred to fresh microcentrifuge tubes after the incubation and set aside until further use.

##### Treatment of Lymphocyte Culture

The cell viability of lymphocytes on PAni–Cs composite samples was evaluated using the same method described in [1]. The assay was conducted using 270 µL of lymphocyte culture. This prepared lymphocyte culture was added to each microcentrifuge tube containing 30 µL of an extract from the composite films (pure Cs, 1:1 PAni–Cs, and 1:10 PAni–Cs), RPMI supplement (positive control), and 0.1% Triton X-100 (negative control). Treated lymphocyte cultures were mixed and incubated with 5% CO_2_ at 37 °C for about 24 ± 3 h.

##### Cell Count

Treated cultures, after 24 h incubation, were collected for cell counting. Prior to cell counting, 7 µL of each treated culture was added to 7 µL of trypan blue. The mixed solutions were placed in a hemocytometer. Cell counting was done in all 25 squares within the 1 mm center grid, where the number of live lymphocytes as well as the number of dead lymphocytes was counted. The cell density (number of cells per mL) was computed using the following equation [1]:

(1) $$\begin{array}{l} \frac{no.\;cells}{mL}=\frac{total\;no.\;of\;cells}{no.\;of\;1mm^{2}\;squares}\times 10^{4}\times original\;dilution*\\ {}*\;dilution\;factor\;=\;2 \end{array}$$

##### Statistical Analysis

The cell viability of lymphocytes on PAni–Cs composites was statistically analyzed using a one-sample test for varying chi-square distribution. The statistical method was done using Originlab 2019 software (OriginLab Corporation, Northhampton, MA, USA) where the null and alternative hypotheses were defined in terms of population variance. The test statistic is calculated using the following equation:

(2) $$\frac{(n-1)s^{2}}{\sigma^{2}},$$

where *n* is the total number of data, *s*^2^ is the sample variance, and σ^2^ is the population variance. The degrees of freedom (df) are *n* − 1. The null hypothesis in this study was accepted when the population variance was equal to the test or sample variance. Otherwise, the hypothesis was rejected.

## 3. Results

### 3.1. PAni Sample Analysis

**Figure 1:**
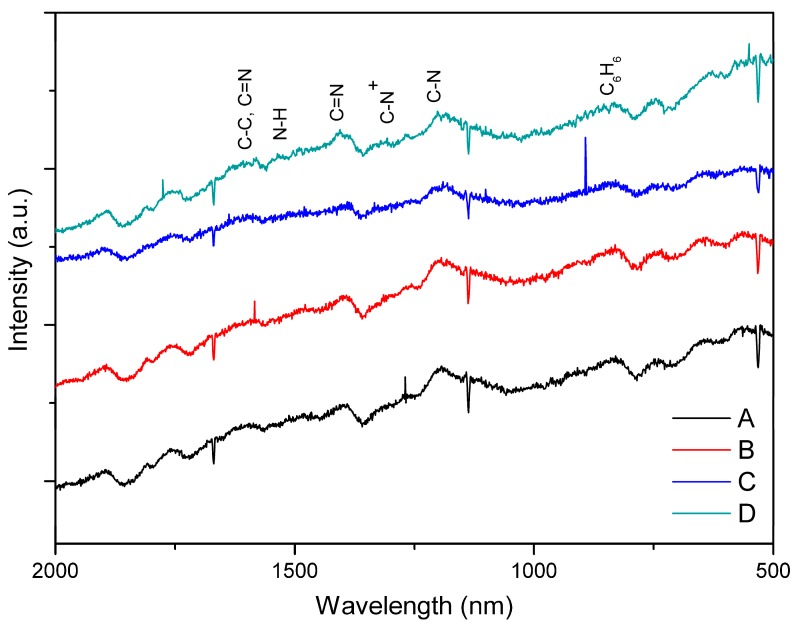
Raman spectra of polyaniline (PAni) powders polymerized under constant aniline (An) concentration (5.48 M) and different concentrations of acid dopants: (**A**) 0.1 M HCl; (**B**) 0.1 M CH_3_COOH; (**C**) 0.01 M HCl; and (**D**) 0.01 M CH_3_COOH. a.u.: Arbitrary units.

Raman spectroscopy was used to confirm the molecular structure of the synthesized PAni powders under different acid dopant concentrations. Figure 1 shows the spectra of PAni samples A, B, C, and D synthesized using 5.48 M of An solution, combined with 0.1 and 0.01 M solutions of HCl and CH_3_COOH, respectively. Characteristic Raman peaks of PAni can be observed within the wavelength range 500–2000 nm [23]. The C=N stretching vibration in the quinonoid units of PAni is observed at 1406 nm. The band at 1200 cm^−1^ represents the C–N stretching vibrations of various benzenoid, quinonoid, and polaronic forms. These polaronic forms lead to the formation of PAni in the emeraldine state. Specifically, these forms are evident with the presence of C–N^+^ vibrations at 1350 nm. The band at 843 nm represents the substituted benzene ring deformations. Bands at ≈1600 and ≈1500 cm^−1^ corresponds to the C–C stretching of the benzenoid ring vibrations and C=N vibration bands, as well as N–H vibration bands of PAni, respectively. Below 1200 nm attributes to the C–H in-plane bending vibrations [23,24]. Based on these results, polymerization of An under different acid dopant concentrations did not yield a drastic effect on the chemical features of PAni, as evidenced by the characteristic peaks observed in all samples. This then indicates that PAni can be synthesized using a weaker acid dopant rather than strong ones such as HCl.

### 3.2. Surface Morphology and Composition of PAni–Cs Films

**Figure 2:**
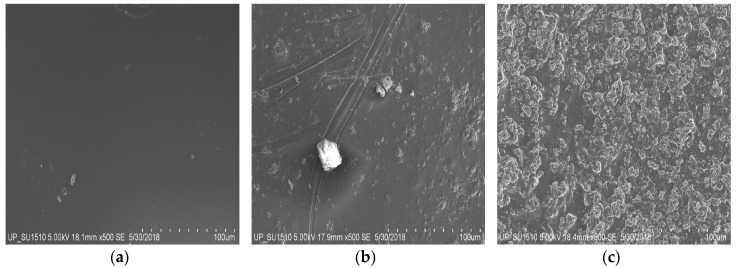
Scanning electron microscopy (SEM) images of polyaniline–chitosan (PAni–Cs) composite films observed at a magnification of 500×, accelerating voltage of 5 kV, and working distance of 18.1 mm. (**a**) Pure chitosan (Cs), (**b**) 1:10 PAni–Cs, and (**c**) 1:1 PAni–Cs.

The morphology of the synthesized pure Cs, 1:10, and 1:1 PAni–Cs composite films was evaluated using SEM–EDX analysis, and the results are shown in Figure 1. The pure Cs film (Figure 2a) revealed a smooth surface feature and no surface defects. The presence of PAni in the composite films, on the other hand, was confirmed by the irregular-shaped particles observed in the micrographs of the 1:10 and 1:1 PAni–Cs composite films (Figure 2b,c). Moreover, the amorphicity of the PAni–Cs composite film was more defined in the 1:1 ratio, which may possibly be due to the agglomeration of PAni particles, with an average size of 2.834 ± 1.029 µm, present on the chitosan surface.

Surface composition of the PAni–Cs films was analyzed using EDX. The wt % of carbon and oxygen for each sample is summarized in Table 2. Pure Cs film had the lowest wt % of carbon. The wt % of carbon in the 1:10 and 1:1 PAni–Cs film samples was 59.249% and 53.056%, respectively. The presence of PAni in these films allowed the samples to have a higher C content. Moreover, the differences in the O wt % content confirmed the addition of PAni into the chitosan mixture, producing the target composite film.

### 3.3. UV–Vis Spectroscopic Study of PAni–Cs Films

**Figure 3:**
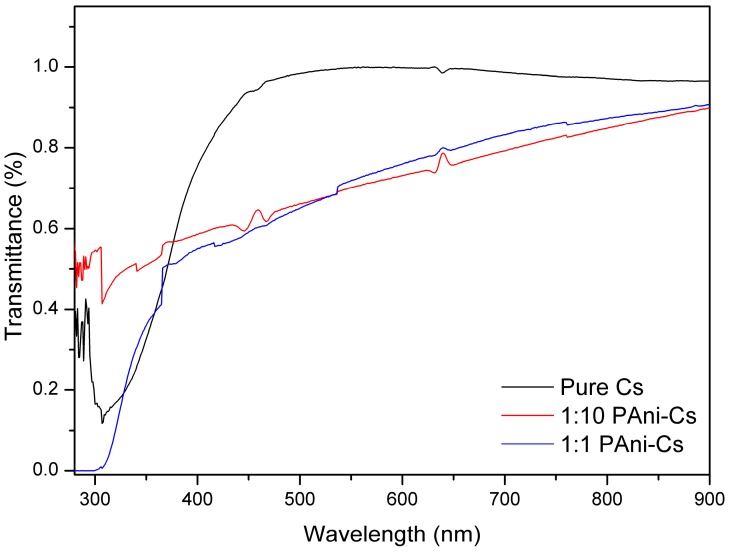
Ultraviolet–visible (UV–Vis) spectra of (**a**) pure chitosan (Cs), (**b**) 1:10 polyaniline–chitosan (PAni–Cs), and (**c**) 1:1 PAni–Cs.

Chitosan has the ability to absorb light at characteristic wavelengths in the UV–Vis region because of its glucosamine units [25]. The UV–Vis spectra of the PAni–Cs composite films, obtained in transmittance mode, are shown in Figure 3. Pure Cs showed a small absorption band at ≈320 nm. This can be attributed to the glucopyranose component of the polymer [10]. Interaction of chitosan with the PAni powders may be observed in the composite films. Changes in the percentage of transmittance values at the 320 nm peak possibly indicate the presence of PAni powders. The percentage of transmittance value at the 400–600 nm range decreased in the spectral lines of 1:10 and 1:1 PAni–Cs composite films. This effect may have been caused by the overlapping π–π* electron transition of the benzenoid segments of PAni to polaron transition [25,26]. In addition, this also suggests that the decrease in the percentage of transmittance value may be due to the transition of quinonoid rings of PAni [26]. Based on these results, the curves for the 1:10 and 1:1 PAni–Cs films indicate a potential to exhibit protonation–deprotonation behavior of PAni when combined with chitosan and acetic acid mixture [26].

### 3.4. SR-FTIR Spectral Analyses of PAni–Cs Composites

**Figure 4:**
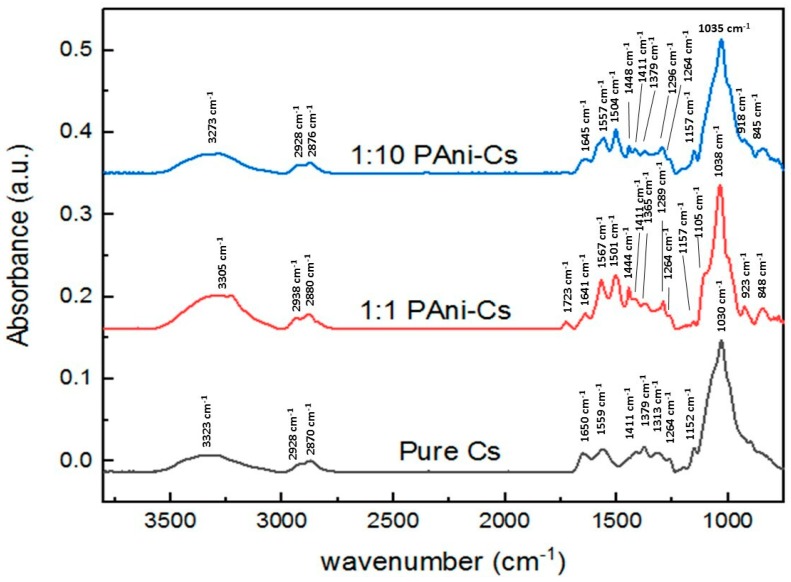
Synchrotron radiation-based Fourier-transform infrared (SR-FTIR) spectra of Pure Cs, 1:10 PAni–Cs, and 1:1 PAni-Cs composites. a.u.: Arbitrary units.

Synchrotron radiation-based Fourier-transform infrared spectra of pure Cs and PAni–Cs composites (with 1:1 and 1:10 ratios) were recorded within the 4000–500 cm^−1^ range (Figure 4). The characteristic peaks of each of the samples are summarized in Table 3. The peak at 3323 cm^−1^ in pure Cs indicates stretching vibrations of N–H in primary amines and O–H in the pyranose ring. Peaks observed at 2928 and 2870 cm^−1^ were due to C–H stretching vibrations of chitosan in the CH_2_OH group and pyranose ring, respectively. The peak at 1650 cm^−1^ was attributed to C=O stretching vibrations in the amide I band, while the peak at 1559 cm^−1^ was due to the N–H bending vibrations of amide II. Stretching vibrations in amide I, II, and III were observed at the 1411 cm^−1^ peak. Also, C–H bending vibrations in the methyl side chain of the amide functional group were assigned at 1379 cm^−1^. The peaks observed at 1313 and 1264 cm^−1^ in the pure Cs spectrum appear to be the C–H bending vibrations in the chitosan ring structure and N–H bending vibrations in the amide group, respectively. Peaks at 1152 and 1030 cm^−1^ were caused by the presence of C–O stretching vibrations in chitosan.

After adding PAni into the Cs mixture, a small shift of peaks was observed both for the 1:1 and 1:10 PAni–Cs composites with N–H stretching vibrations at 3305 and 3273 cm^−1^, C–H stretching vibrations at 2938/2880 and 2924/2876 cm^−1^, respectively. A slight shift of peaks was also detected with the amide I and II vibrations at 1723–1641 and 1654 cm^−1^ for PAni–Cs composites. Small shifts were further observed on the C–H bending vibration of the amide methyl group (1365 cm^−1^), and for the C–O stretching vibrations in chitosan at 1157–1038 cm^−1^ (1:1 PAni–Cs) and 1157–1035 cm^−1^ (1:10 PAni–Cs). These minor shifts of peak position were due to some conformational changes and interactions between chitosan and polyaniline [27].

The characteristic absorption bands of PAni were also observed in the 1:1 and 1:10 PAni–Cs spectra. Specifically, the absorption peaks at 1567/1557 and 1501/1504 cm^−1^, respectively, corresponded to the C=N stretching vibration of the quinonoid ring and the C=C stretching vibration of the benzenoid ring unit. The presence of these peaks suggests that the synthesized composite samples contained PAni. In addition, the oxidation state of PAni in the samples was determined according to the integrated intensity peaks of the quinonoid (C=N stretching vibrations) and benzenoid (C=C stretching vibrations) units. The degree of oxidation was related to the ratio of the quinonoid and benzenoid units in the PAni backbone. Using the values obtained in the integration of these peaks, the degrees of oxidation for 1:1 and 1:10 PAni–Cs composites were calculated to be 51.07% and 50.87%, respectively. These values showed almost equal occurrence of the quinonoid and benzenoid units in the polyaniline of PAni–Cs samples. The calculated degree of oxidation in the PAni–Cs samples confirms that the PAni in the samples were in an emeraldine state. The peak at 1289/1296 cm^−1^ refers to the C–N stretching vibration of the benzenoid unit. The presence of this peak further indicates that there was an occurrence of π-electron delocalization in the polymer chain due to the protonation process during polymerization [28]. The characteristic band observed at 1444/1448 cm^−1^ corresponds to the C=C and N=N stretching vibrations and the C–H bending vibration in the benzenoid ring of the PAni structure, respectively. The appearance of a peak at 848/845 cm^−1^ was caused by the aromatic ring out of plane deformation vibrations and particularly because of the C–H outer plane bending of the benzenoid ring [29,30]. Moreover, vibrations in the pyranose ring of chitosan were detected at 923/918 cm^−1^ for the PAni–Cs samples. 

**Figure 5:**
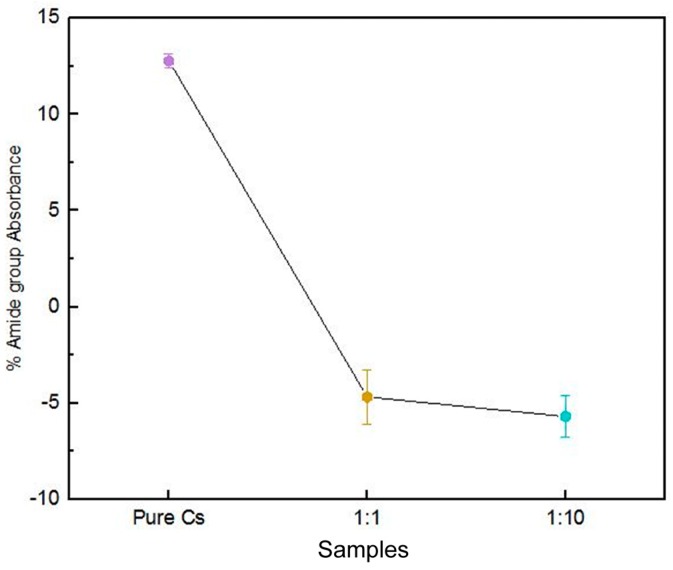
Amount of amide present in pure chitosan (Cs), 1:10 polyaniline–chitosan (PAni–Cs), and 1:1 PAni–Cs composites.

When polyaniline and chitosan are mixed together, shifts in the characteristic peaks of their SR-FTIR spectrum can show whether chemical interactions happen between them. In the spectra of the PAni–Cs composite films, the C=O and N–H vibrations in the amide group band of chitosan shifted from 1650 to 1723 cm^−1^, and from 1559 to 1641/1645 cm^−1^, respectively. The shift of the amide band of chitosan to a higher frequency may be expected since this can result from the addition of polyaniline where the polymer may have caused weakening of the hydrogen bond interactions between the molecules of the two polymers. The difference in the composition of the films can be distinguished by the spectral shifts of the amide bands, since changes in the hydrogen bond strength are related to the concentration of the substances [31]. Figure 5 shows a graphical representation of the amount of amide present in terms of its percentage of absorbance. This representation was determined by calculating the area under curves of each characteristic SR-FTIR peak (specifically for the amide I and II groups) via integration. It was observed that a decrease in the percentage of absorbance of amide bands corresponds to a decrease in its concentration in the composites.

**Figure 6:**
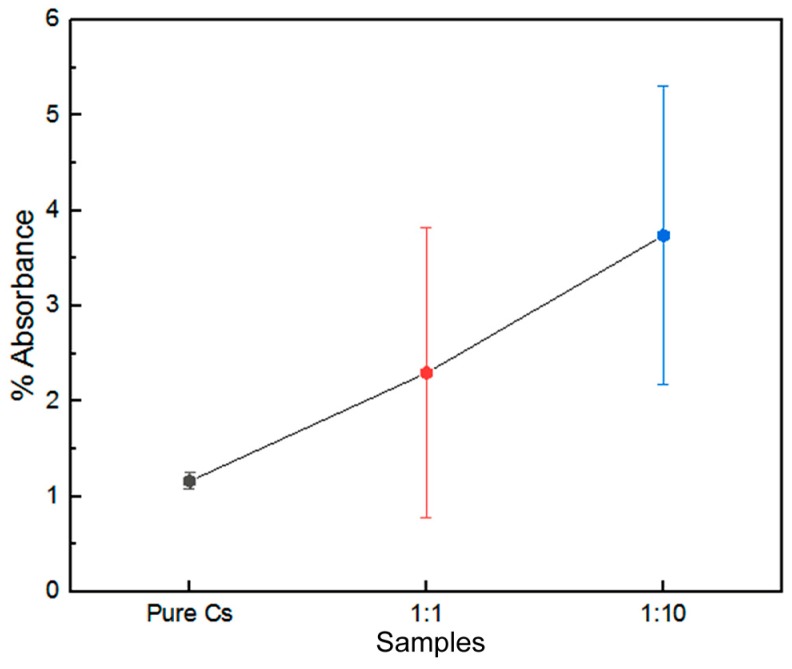
Absorbance of the C–H stretching bands present in pure chitosan (Cs), 1:10 polyaniline–chitosan (PAni–Cs), and 1:1 PAni–Cs composites.

In addition, the absorption band at 1313 cm^−1^ (C–H bending vibration of chitosan) shifted to a lower frequency at 1289/1296 cm^−1^ for the PAni–Cs composites. This minor shift to a lower frequency was recognized by the addition of polyaniline to chitosan, and it may be attributed to the C–N stretching vibration in the benzenoid ring of PAni. Figure 6 shows a graphical representation of the percentage of absorbance at peaks 1313, 1289, and 1296 cm^−1^. In the graph, the increase in percentage of absorbance was caused by the presence of PAni chains in the composite film.

Generally, the SR-FTIR spectra of the samples produced through a solution casting method indicate that the interactions between chitosan and polyaniline resulted in the shifting of characteristic peak positions.

### 3.5. Cell Viability Assessment

**Figure 7:**
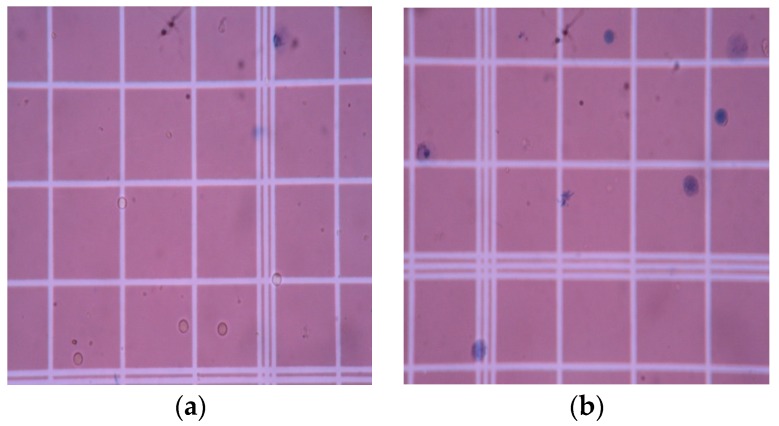
Cytocompatibility assay of (**a**) positive and (**b**) negative controls, supplemented RPMI and 0.1% Triton X-100, respectively.

**Figure 8:**
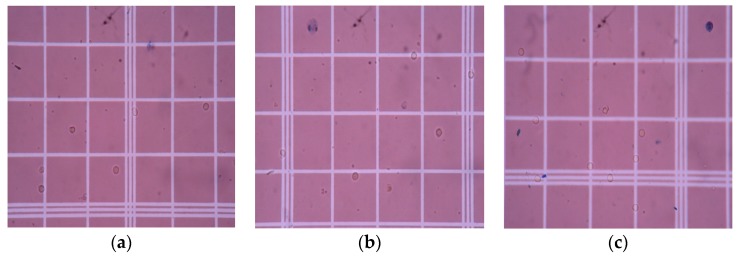
Cytocompatibility assay of (**a**) pure chitosan (Cs), (**b**) 1:10 polyaniline–chitosan (PAni–Cs), and (**c**) 1:1 PAni–Cs.

**Figure 9:**
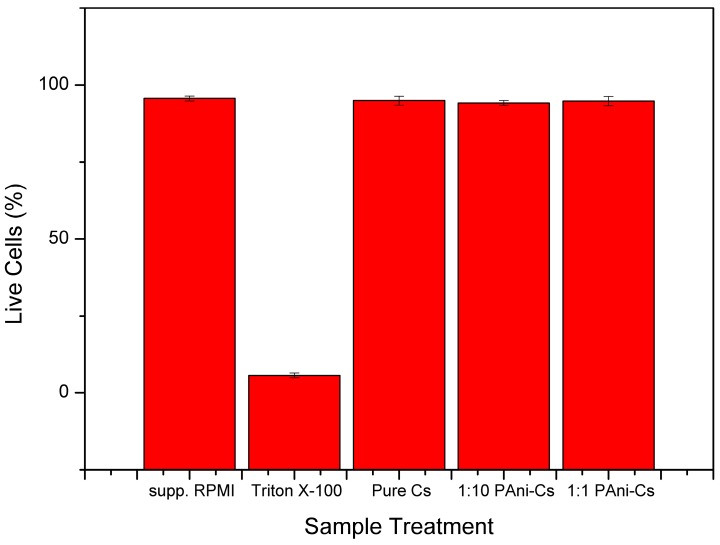
Cell viability of the supplemented RPMI (positive) and Triton X-100 (negative) and the polyaniline–chitosan (PAni–Cs) composite films.

The effect of PAni on the biocompatibility of chitosan was assessed using a trypan blue assay. Live lymphocyte cells have intact membranes and will show colorless cytoplasm under a microscope, while dead cells have damaged membranes that are penetrated by the dye and will show a blue cytoplasm [1]. The results of the cell viability assay can be observed in Figure 7 and Figure 8, while the total percentage of live cells, calculated using Equation (1), is shown in Figure 9. The assay revealed that lymphocytes treated with RPMI supplement and 0.1% Triton X-100 showed a cell viability of 95.63 ± 0.855% and 5.67 ± 0.8%, respectively. On the other hand, the cell viability results of the PAni–Cs composite films revealed that pure Cs, and 1:10 and 1:1 PAni–Cs film samples yielded a total percentage of live cell values of 94.93 ± 1.5%, 94.17 ± 0.76%, and 94.77 ± 1.51%, respectively. These values indicate that the films were highly viable and comparable with the positive control (RPMI-supplemented media).

A chi-square one-sample variance test was calculated to test the homogeneity of variances for the percentage of total live cells in each treatment. Originlab 2019 was used for this test statistic and results are summarized in Table 4. The calculated population variances of pure Cs, and 1:1 and 1:10 PAni–Cs were 2.25660, 2.33754, and 0.5730, respectively. These calculated values, at a 0.05 level of significance (α), imply that the population variance of pure Cs was not significantly different from the variance test, which was 2.25333. Variance tests for both 1:1 PAni–Cs and 1:10 PAni–Cs samples did not yield significant difference from their corresponding variance test values of 2.29333 and 0.57333, respectively. These calculations were supported by a chi-square distribution, which compared the *p*-values of each sample at the 0.05 α-level. Table 4 shows that α was less than the computed *p*-values for pure Cs and 1:1 and 1:10 PAni–Cs. This means that there were no significant differences between the population variance and the variance test of sample treatments. Therefore, statistical analysis reveals that the percentage of total live cells of pure Cs did not yield significant difference with values for 1:10 and 1:1 PAni–Cs samples, implying that all samples are highly viable.

Based on these results, it can be surmised that synthesized PAni–Cs composites are nontoxic and can possibly be used for biomedical applications. However, further evaluation of the biocompatibility of this composite is suggested at various contact times.

## 4. Discussion

Polyaniline powders were polymerized from An solution mixed with varying concentrations of HCl and CH_3_COOH acid dopant media. The chemical oxidative polymerization of PAni powders is usually performed with HCl, a very strong acid, as the acid dopant. However, PAni exhibits poor biocompatibility, which may be due to the influence of HCl. Thus, this study explored the feasibility of polymerizing An under a weaker acid environment. Raman spectra analysis revealed that the PAni powders synthesized using high and low concentrations of HCl and CH_3_COOH had no significant difference with each other. This implied that the characteristic peaks of PAni were evident in all sample treatments. However, further investigation may still be needed to determine the effects of weaker acid dopants on the morphological and conductivity properties of PAni powders.

The synthesized PAni powders, specifically sample D, were used to fabricate a PAni–Cs film using a solvent casting approach. The addition of PAni powders was observed on the morphology of the film samples where the amorphicity of the composites increased due to the agglomeration of PAni powders on the chitosan surface. Moreover, the structures of the PAni–Cs films were investigated using UV–Vis spectroscopy. The UV–Vis spectra confirmed the chemical structure and overlapping electronic transitions as well as the protonation behavior of PAni in the PAni–Cs composite films. The chemical interactions were also observed in the SR-FTIR spectra of the composites, where shifting of characteristic peak positions of chitosan was observed in the 1:10 and 1:1 PAni–Cs samples. Further analysis on the SR-FTIR spectra of the PAni–Cs composites confirmed that PAni in these samples was in an emeraldine state.

A cell viability assay was also conducted to determine the possible effect of PAni on the biocompatibility of chitosan. As mentioned, conducting polymers such as PAni are cytotoxic possibly due to the presence of impurities or the acid dopant used [15]. In this study, PAni powders were polymerized using a low concentrated CH_3_COOH acid dopant medium. Results of the cell viability assay showed that incorporating PAni into a chitosan mixture may improve the biocompatibility of PAni for possible biomedical application, such as tissue engineering. Statistical analysis revealed that the percentage of total live cells yielded in 1:1 and 1:10 PAni–Cs samples did not significantly differ with the percentage of total live cells of pure Cs that are cell viable. Moreover, the assay may indicate that the PAni and the acetic acid concentrations utilized in this study were not harmful to living cells as manifested by the high cell viability shown in Figure 9. This study, therefore, shows that PAni–Cs composites may be further explored in the biomedical field to maximize the potential of these two polymers.

## 5. Conclusions

This study showed that PAni powders may be formed using CH_3_COOH as the acid dopant medium, aside from HCl. The synthesized PAni powders exhibited the Raman characteristic peaks of a synthetic polymer, implying that the acid dopant used did not drastically affect the chemical features of the powder. Polyaniline–chitosan composites were also synthesized using a solution casting method. The presence of PAni powders on the composite was confirmed using SEM–EDX equipment, which showed agglomerated particles on the surface of the composites due to the presence of the PAni particles. Moreover, analysis of the UV–Vis and SR-FTIR spectra observed the interaction of the two polymers, polyaniline and chitosan, on the composite samples. The presence of PAni powders caused changes in the percentage of transmittance values of the 1:10 PAni–Cs and 1:1 PAni–Cs samples at the 320 and 400–600 nm characteristic peaks. Furthermore, the chemical structure of PAni in the PAni–Cs composites were identified due to the benzenoid and quinonoid characteristic absorbance bands observed in 1:10 and 1:1 PAni–Cs composite films. Cell viability assays revealed that the PAni powders did not affect the biocompatibility of chitosan, indicating that PAni–Cs composite films may possibly be utilized for future biomedical application.

## Figures and Tables

**Table 1:** Polyaniline samples with different aniline and acid dopant concentration ratios.

Sample	Aniline to Acid Dopant Concentration Ratio
A	5.48 M aniline:0.1 M HCl
B	5.48 M aniline:0.1 M CH_3_COOH
C	5.48 M aniline:0.01 M HCl
D	5.48 M aniline:0.01 M CH_3_COOH

**Table 2:** Composition of pure chitosan (Cs) and 1:10 and 1:1 polyaniline–chitosan (PAni–Cs) composites obtained using energy-dispersive X-ray spectroscopy (EDX).

% Weight	Pure Cs	1:10 PAni–Cs	1:1 PAni–Cs
C	37.505	59.249	53.056
O	41.419	40.751	46.944

**Table 3:** Characteristic absorption bands in the synchrotron radiation-based Fourier-transform infrared (SR-FTIR) spectra of pure Cs, 1:10 PAni–Cs, and 1:1 PAni–Cs composites.

Wavenumber (cm^−1^)			Vibration Modes
Pure Cs	1:1 PAni–Cs	1:10 PAni–Cs	
3323	3305	3273	ν(NH_2_) in primary amines ν(OH) in pyranose ring
2928	2938	2924	ν(CH_2_) in CH_2_OH group
2870	2880	2876	ν(C–H) in pyranose ring
1650–1559	1723–1641	1645	ν(C=O) in amide I band δ(N–H) of amide II
-	1567	1557	ν(C=N) of quinoid ring
-	1501	1504	ν(C=C) of benzenoid ring
-	1444	1448	ν(C=C) of aromatic ring ν(N=N) in PAni structure
1411	1411	1411	ν- vibrations in amide I, II, and III
1379	1365	1379	δ(C–H) in methyl group of amide
1313	1289	1296	δ(C–H) in chitosan ring structure ν(C–N) of benzenoid ring
1264	1264	1264	δ(N–H) in amide group
1152	1157	1157	ν(C–O) in glycosidic linkage
-	1105 (shoulder)	-	ν(C–O) in glycosidic linkage
1030	1038	1035	ν(C–O) in secondary OH group
-	923	918	vibrations in pyranose ring
-	848	845	δ(C–H) of benzenoid ring

**Table 4:** One-sample variance test for the percentage of total live cells of each sample treatments: pure chitosan (Cs), 1:1 polyaniline–chitosan (PAni–Cs) and 1:10 PAni–Cs.

Descriptive Statistics					Test Statistics		
	*N*	Mean	SD	Variance	Chi-Square	df	*p*-value
Pure Cs	3	94.93333	1.50111	2.25333	4.50667	2	0.2101
1:1	3	94.76667	1.51438	2.29333	4.58667	2	0.20186
1:10	3	94.16667	0.75719	0.57333	1.14667	2	0.87271

df: Degrees of freedom; SD: Standard deviatioin.
